# A Proliferation-Inducing Ligand Regulation in Polymorphonuclear Neutrophils by *Panax ginseng*

**DOI:** 10.1007/s00005-020-00597-z

**Published:** 2020-10-13

**Authors:** Wioletta Ratajczak-Wrona, Natalia Wawrusiewicz-Kurylonek, Marzena Garley, Adam Jacek Kretowski, Ewa Jablonska

**Affiliations:** 1 Department of Immunology, Medical University of Bialystok, J. Waszyngtona 15A, 15-269 Bialystok, Poland; 2 Department of Endocrinology, Diabetology and Internal Medicine, Medical University of Bialystok, Bialystok, Poland; 3 Clinical Research Centre, Medical University of Bialystok, Bialystok, Poland

**Keywords:** APRIL, TGF-β, Ginsenoside Rb1, Neutrophils, PI3K/Akt, p38

## Abstract

A proliferation-inducing ligand (APRIL) is a member of the tumor necrosis factor superfamily that was first identified as a factor favoring tumorigenesis. APRIL is important fitness and survival factors for B cells and plasma cells in the periphery. Considering this, as well as the quantitative predominance of neutrophils among the peripheral blood leukocytes, we carried out the first study assessing the influence of the transforming growth factor (TGF)-β signaling pathway on APRIL expression in these cells. Furthermore, as the Rb1 ginsenoside is known to exhibit multiple pharmacological activities, we verified if the saponin is capable of modulating the process. The present study shows that TGF-β increased the expression of APRIL and the level of phospho-p38, phospho-Akt(T308), and phospho-Akt(S473) in the cytoplasmic fraction, as well as the expression of Fra1, c-Fos, and c-Jun in the nuclear fraction, of neutrophils. However, exposure of these cells to Rb1 reduced the expression and level of the investigated proteins. No changes were found in the expression of APRIL and the level of p-p38 in the cytoplasmic fraction of neutrophils following the application of Rb1 alone, as well as in the neutrophils incubated first with Rb1 and then with TGF-β, whereas a higher level of phosphorylation was observed for Akt and PI3 kinases in the cells. Moreover, a higher expression of all the studied transcription factors was observed in the nuclear fraction of neutrophils. Based on the observed changes, it may be assumed that the expression of APRIL molecule in TGF-β-induced neutrophils and its regulation by Rb1 are associated with PI3K/AKT signaling pathways and transcription factors Fra-1, Fra-2, c-Jun, and c-Fos. Rb1 appears to be a favorable factor that may be potentially used in the modulation of tumor-promoting APRIL expression.

## Introduction

Ginseng is the root of *Panax ginseng* C.A. Meyer (Araliaceae family). It has long been used in Asia, particularly in China, as a therapeutic herb for improving the well-being and alleviating tiredness (Kang and Min 2012). The main bioactive components of ginseng are tetracyclic triterpenoid saponins (ginsenosides), including Rb1, Rg1, Rc, and Rg3 (Chen et al. 2019; Zhuang et al. 2014). They are attributed with properties such as neuroprotectve activity, which can be utilized in the treatment of neurodegenerative diseases (Kang et al. 2019; Li et al. 2019). Animal and cell culture studies have also shown the anticancer and metastatic effects of individual *Panax ginseng* compounds through their influence on angiogenesis, proliferation, apoptosis, and telomerase activity in cancer cells. Specific anticancer activity is attributed to ginsenosides Rg5, Rg3, Rh2, and Rb1 (Kang and Min 2012; Lee et al. 2014; Skopinska-Rozewska 2009).

Ginsenoside Rb1 constitutes the highest amount in ginseng. It was found that this saponin shows estrogenic activity by activating the α and β receptors of estrogens (Cho et al. 2004). Rb1 may be transformed into an active metabolite K as a result of the action of intestinal bacteria (Kang and Min 2012; Park et al. 2005). Metabolite K suppresses the production of PGE2, reduces the synthesis of nitric oxide, and inhibits the expression of NF-κB in macrophages stimulated with lipopolysaccharide (LPS). At high concentrations, metabolite K, in contrast to Rb1, increases the level of COX2 (Park et al. 2005). This saponin has also been shown to increase the level of Treg and Th lymphocytes, increase antibody-dependent cytotoxicity, and activate NK cells (Kang and Min 2012; Park et al. 2005). While inhibiting the expression of adhesion molecules, ICAM-1 and VCAM, it reduces the activation and adhesion of monocytes to endothelial cells (Wang et al. 2011). Moreover, ginsenoside Rb1 inhibits apoptosis in damaged nerve cells and cardiomyocytes by increasing the antiapoptotic expression of Bcl-2 protein and decreasing the proapoptotic expression of Bax protein (Guan et al. 2002; Kenarova et al. 1990; Scaglione et al. 1990; See et al. 1997).

The literature data emphasize the important role that ginsenoside Rb1 plays in the modulation of the immune response, for example, by acting on innate immune cells, including neutrophils (Sun et al. 2007; Youn and Gabrilovich 2013).

Neutrophils are the most abundant circulating leukocytes and are critical effector cells of the innate immune system (Kobayashi 2015; Rosales 2018). Their role in carcinogenesis, however, is still under deliberation. Although polymorphonuclear neutrophils are traditionally considered antitumoral in the context of their anti-bacterial functions, it is becoming increasingly clear that tumor-associated neutrophils (TANs) play a major role in cancer biology (Sionov et al. 2015; Wang et al. 2018). Recent studies suggest that the presence of abundant neutrophils within a tumor is associated with increased tumor growth and hence a poor prognosis (Fridlender and Albelda 2012; Granot and Fridlender 2015; Treffers et al. 2016). Fridlender et al. (2009) characterized TANs into two subcategories of N1 and N2. N1 neutrophils are characterized by high production of pro-inflammatory cytokines and chemokines, an hypersegmented nuclei, low levels of arginase and, in vitro*,* can eliminate increased numbers of tumor cells. In contrast, N2 subtype have strong immunosuppressive and tumor promoting activity (Andzinski et al. 2015; Coffelt et al. 2016). TANs have been shown to be capable of polarization into either an anti-tumorigenic “N1” phenotype by type-1 interferons or a pro-tumorigenic (N2) phenotype by the presence of transforming growth factor (TGF)-β (Shaul et al. 2016).

TGF-β is a pleiotropic cytokine, which is produced by numerous cells. It plays a significant role in maintaining tissue homeostasis, among others, by regulating the processes of growth, differentiation, cell migration, formation and degradation of extracellular matrix components, chemotaxis, and apoptosis (Cantelli et al. 2017; Yang et al. 2010). The effect of TGF-β in the cancerous process is referred to as the “TGF-β paradox” (Tian and Schiemann 2009). TGF-β has been demonstrated to act as a cancer suppressor during the early stages of cancer. However, in the advanced stage, it acts as a tumor promoter, contributing to the growth, invasion, and metastases of tumors. Certain cancers most likely develop TGF-β-inactivating mutations and progress independently of this cytokine. However, others have mutations in cancer suppressor genes that participate in the TGF-β signaling pathway (Tian and Schiemann 2009; Yang et al. 2010; Zhang et al. 2014).

The Smad2/3-dependent pathway is assumed to be the key TGF-β signaling pathway (Wrana 2019). It conducts the signals from kinase type II receptor (TβR-II), through kinase type I receptor (TβR-I), to effector proteins, which then move to the cell nucleus and affect the transcription of target genes (Attisano and Wrana 2002; Cantelli et al. 2017). Amplification of TGF-β signaling may also occur via an alternative course (Smad2/3-independent), involving small GTPases (RhoA, PKN, and Rock), MAP kinases, and PI3 kinase. All these may bind and mediate the Smad-dependent signaling or may constitute a completely distinct signaling pathway in different types of cells (Derynck and Zhang 2003; de Caestecker 2004; Keski-Oja et al. 2004; Mehra and Wrana 2002; Miyazawa et al. 2002; Shi and Massague 2003; Zhu and Burgess 2001). Data suggest that this cytokine modulates the synthesis and expression of the proliferation-inducing ligand APRIL in mouse macrophages (Jang et al. 2011).

A proliferation-inducing ligand (APRIL) is a member of the tumor necrosis factor (TNF) family and consists of 250 amino acids encoded by a gene located at chromosome 17p13. APRIL is produced mostly by cells from the myeloid lineage, including monocytes/macrophages, neutrophils, and eosinophils (Dillon et al. 2006; Vincent et al. 2013a). APRIL signal transduction is mediated by direct binding of the cytokine to at least two cell–surface receptors: transmembrane activator and calcium-modulator and cyclophilin ligand interactor, and B cell maturation antigen (Bat-Erdene et al. 2018; Vincent et al. 2013b; Wollacott et al. 2019). APRIL has antagonistic properties when compared with most of TNF family members. This protein is one of the few that have the ability to stimulate cell growth in different cancer lines (Vincent et al. 2013b).

APRIL is also expressed in cells outside the immune system, including osteoclasts and tumor tissues (Hahne et al. 1998). In cancer patients, a high level of APRIL expression indicates a shortened survival time due to the increased aggressiveness of the disease. Studies have shown that APRIL is involved in the induction of signals leading to the proliferation of both leukemia B cells and solid cancer cells (He et al. 2004; Mhawech-Fauceglia et al. 2006). An increased expression of APRIL is observed in Hodgkin’s lymphoma, non-Hodgkin’s lymphoma, chronic lymphocytic leukemia, and multiple myeloma, as well as in solid intestinal, pancreatic, and breast tumors (Hahne et al. 1998). Data have been reported that cancer cells and hematopoietic cells including infiltrating neutrophils are the source of APRIL in the course of cancer (Mhawech-Fauceglia et al. 2006; Moreaux et al. 2009).

Neutrophils remain in the peripheral blood for approximately 8 h and then reach the tissues where they survive for up to 3 days (Keshavan et al. 2019; Pillay et al. 2010). Similar to almost all cells found in an organism, neutrophils also possess TGF-β receptors (Brandes et al. 1991; Shen et al. 2007). However, only a few studies analyzing the TGF-β pathway in neutrophils are available in the literature, and these do not provide any information on the impact of TGF-β on APRIL expression (Shen et al. 2007; Travis and Sheppard 2014). Considering this, as well as the quantitative predominance of neutrophils among the peripheral blood leukocytes, we carried out the first study assessing the influence of the TGF-β signaling pathway on APRIL expression in these cells. Furthermore, as the Rb1 ginsenoside is known to exhibit multiple pharmacological activities (Xin et al. 2019), we verified if the saponin is capable of modulating the process. Understanding the mechanisms that regulate the expression of APRIL in neutrophils can contribute to analyzing the general mechanisms of signal transduction in these cells.

## Materials and Methods

### Neutrophil Isolation

Neutrophils were obtained from the blood of 15 healthy volunteers (males). All experimental procedures were approved by the Ethics Committee of the Medical University of Bialystok.

Neutrophils were isolated using density-gradient centrifugation with Polymorphprep™ reagent (AXIS-SHIELD PoC AS, Oslo, Norway). Bürker chamber and Türk’s solution were used for counting the cells. Cell purity was assessed by applying the thick drop method, using May-Grünwald and Giemsa dyes. For subsequent isolation, donor cell preparations that exhibited high cell purity with more than 85% neutrophils were used. To obtain a pure cell fraction, the magnetic MACS^®^ Separator, as well as antibodies and magnetic CD16 MicroBeads (no. catalog no. 130-045-701; Miltenyi Biotec), was used for positive separation. The viability of neutrophils was assessed in preparations formed directly after isolation, as well as in those incubated for 20 h, using trypan blue dye. Sera were obtained from blood samples collected without anti-coagulation agents.

### Neutrophil Incubation

**Fig. 1:**
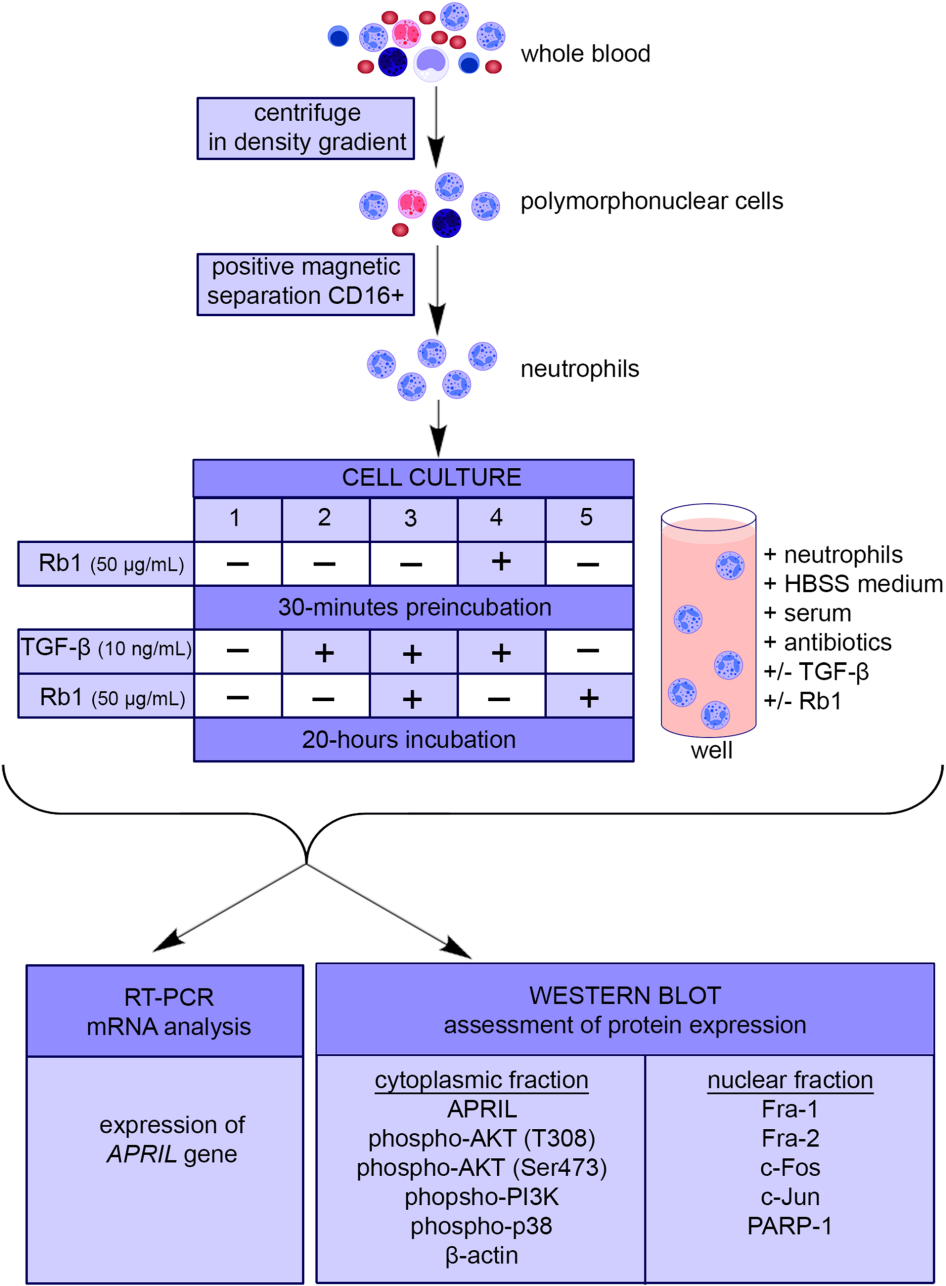
Research methodology workflow

Neutrophils were resuspended in HBSS (Invitrogen, Carlsbad, CA, USA) medium supplemented with donors’ own serum (7.4%), antibiotics (streptomycin and penicillin) and seeded at 5 × 10^5^ cells/well in sterile plates. Neutrophils were divided into five different groups which were incubated with or without human recombinant TGF-β (rhTGF-β; 10 ng/ml) (R&D Systems) and/or ginsenoside Rb1 (50 µg/ml) (HWIANALITIK GMBH Pharma Solution Ruelzheim, Germany) according to cell culture outline presented on Fig. 1. All cells were preincubated for 30 min with or without ginsenoside Rb1. Thereafter rhTGF-β and/or ginsenoside Rb1 were added into wells and the plate was incubated for next 20 h at 37 °C in 5% CO_2_ (Nuaire™ US Autoflow, Plymouth, MN, USA).

### Gene Expression

The whole cellular RNA was extracted and purified using the RNeasy Mini Kit (Qiagen, Germany) by the common protocol. The expression of *APRIL* gene was assessed by two-step quantitative real-time PCR, using ready-to-use TaqMan Gene Expression Assays (Hs00601664_g1, NM_003808.3, ThrmoFisher Scientific, USA) and using the 7900HT Fast Real-Time PCR System (Applied Biosystems, USA). cDNA synthesis was performed using High Capacity cDNA Reverse Transcription Kit (Life Technologies, USA) from 1 µg of total RNA in the MJ Research Thermal Cycler (Model PTC-200, USA). The *18S rRNA* gene was used as a reference gene for normalising qRT-PCR data. The reaction mix consisted of cDNA probes, TaqMan Gene Expression Master Mix (Applied Biosystem, USA) and each TaqMan Gene Expression Assay in the appropriate volume in duplicates. The relative gene expression results were determined by RQ Manager Software (Applied Biosystem, USA).

### Immunoblotting

The collected neutrophils were centrifuged and washed with PBS (Gibco, 10010-015). Cytoplasmic and nuclear protein fractions from these cells were prepared using the NucBuster™ Protein Extraction Kit (Novagen^®^, 71183-3). The protein concentration was detected using a Qubit™ Protein Assay Kit and Qubit 2.0 Fluorometer (Invitrogen, Q33211).

The immunoblotting confirmed the presence of APRIL, phospho-Akt (T308), phospho-Akt (Ser 473), phospho-PI3K, phospho-p38, Fra-1, Fra-2, c-Fos and c-Jun proteins. As an internal control the PARP-1 (nuclear protein fractions) and β-actin (cytoplasmic protein fractions) proteins were used.

Cytoplasmic or nuclear protein fractions were suspended in Laemmli Sample Buffer (Bio-Rad Laboratories, 161-0737) with βME (Bio-Rad Laboratories, 161-0710). Equal amount of proteins was separated by SDS-PAGE and transferred onto nitrocellulose membrane (Bio-Rad Laboratories Mini-PROTEAN® Tetra Cell). Membrane was blocked with 1xTBS 1% Casein Blocker (Bio-Rad Laboratories, 1610782) in the Millipore SNAP i.d.™ Protein Detection System during a 20-s incubation and incubated with primary antibodies 10 min and after that with secondary antibodies 10 min, at room temperature (according to the manufacturer’s instructions).

The proteins were immunoblotted with primary antibodies: anti-human APRIL/TNFSF13 Antibody (R&D Systems, AF884, goat polyclonal; 0.2 μg/ml), p-Akt1/2/3 (B-5) (SantaCruz Biotechnology, sc-271966, mouse monoclonal; 1:100), p-Akt1/2/3 (Ser473) (SantaCruz Biotechnology, sc-33437, rabbit polyclonal; 1:200), p-PI 3-kinase p85α (Tyr508) (SantaCruz Biotechnology, sc-12929, goat polyclonal; 1:200), p-p38 (E-1) (SantaCruz Biotechnology, sc-166182, mouse monoclonal; 1:100), Fra-1 (C-12) (SantaCruz Biotechnology, sc-28310, mouse monoclonal; 1:100), Fra-2 (G-5) (SantaCruz Biotechnology, sc-166102, mouse monoclonal; 1:100), cFos (C-10) (SantaCruz Biotechnology, sc-271243, mouse monoclonal; 1:100), c-Jun (G-4) (SantaCruz Biotechnology, 74543, mouse monoclonal; 1:100), PARP-1 (Ab-3) Mouse mAb (F1-23) (Calbiochem, AM68, mouse monoclonal; 1:5000), β-Actin (9) (SantaCruz Biotechnology, sc-130301; mouse monoclonal; 1:200) followed by the secondary antibodies: Goat Anti-Rabbit IgG-AP (Santa Cruz Biotechnology, sc-2007; 1:5000), Alkaline Phosphatase-conjugated AffiniPure Mouse Anti-Goat IgG (H + L) (Jackson ImmunoResearch Laboratories, 205-055-108; 1:5000) or Alkaline Phosphatase-conjugated AffiniPure Goat Anti-Mouse IgG (H+L) (Jackson ImmunoResearch Laboratories, 115-055-062; 1:5000). Excess unbound antibodies were washed away with TBS-T buffer [10xTBS (Bio-Rad Laboratories, 170-6435) with Tween^®^-20 (Sigma, P9416)]. Detection was performed using the 5-bromo-4-chloro-3-indolylphosphosphate as a substrate, and nitroblue tetrazolium as a chromogenic indicator [BCIP^®^/NBT Liquid Substrate System (Sigma, B1911)].

The color intensity of the protein bands was evaluated by densitometric analysis using ImageJ software (NIH, Bethesda, MD, USA) and presented in graphs as an arbitrary units.

### Statistical Analysis

The data were treated using the STATISTICA version 13.1 program (StatSoft, Inc., Tulsa, OK, USA). The level of statistical significance was preset to *p* < 0.05 (two-tailed). Statistical significance of differences was determined by Student’s *t* test or analysis of variance (ANOVA; Tukey–Kramer’s post-hoc test). Mean values are expressed ± standard error (SE).

## Results

### Evaluation of APRIL mRNA Expression by RT-PCR

**Fig. 2:**
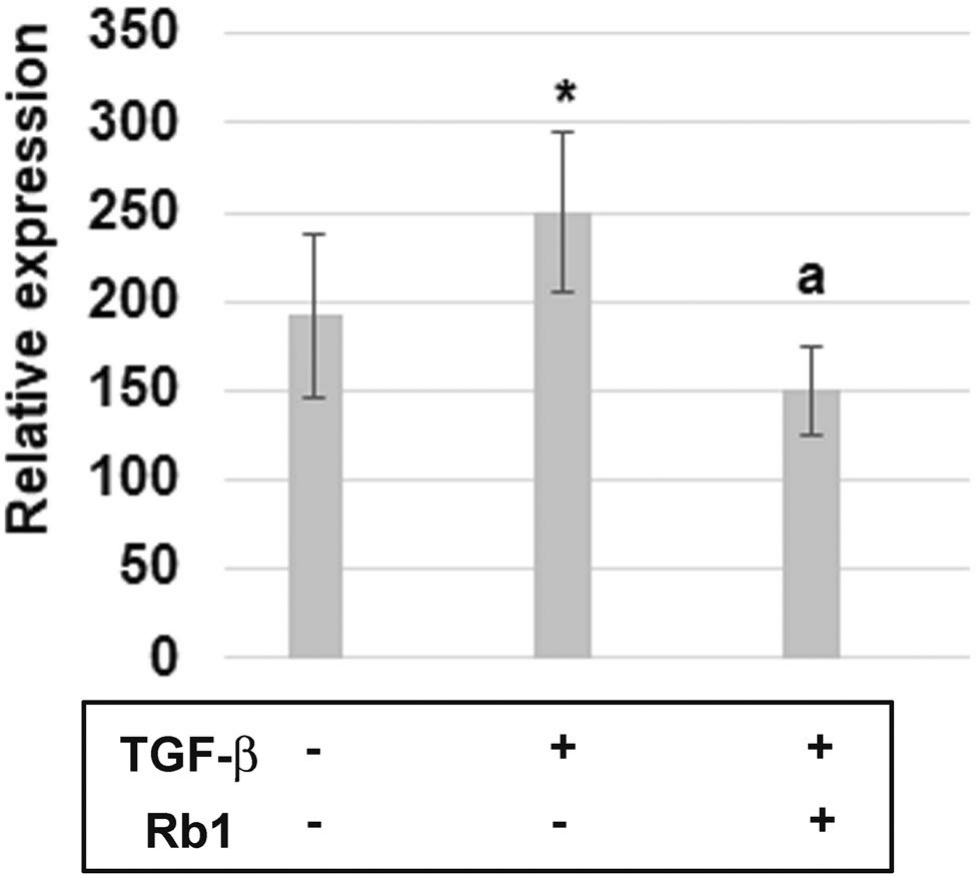
Expressions of APRIL-mRNA in neutrophils from 15 healthy volunteers. Effect of TGF-β (10 ng/ml) or with Rb1 (50 µg/ml) on the expression of APRIL-mRNA. RT-PCR was performed on human neutrophils. mRNA of APRIL levels were normalized to house-keeping gene—s18. Value significantly different between * unstimulated and stimulated cells (p < 0.05); **a** cells incubated only with TGF-β and cells incubated with TGF-β and Rb1 (p < 0.05)

The neutrophils stimulated with TGF-β showed an increased expression of APRIL compared to the nonstimulated cells. However, the neutrophils incubated first with TGF-β and then with Rb1 showed a lower expression of APRIL mRNA compared to the cells stimulated with TGF-β only (Fig. 2).

### Evaluation of Protein Expression by Immunoblotting

**Fig. 3:**
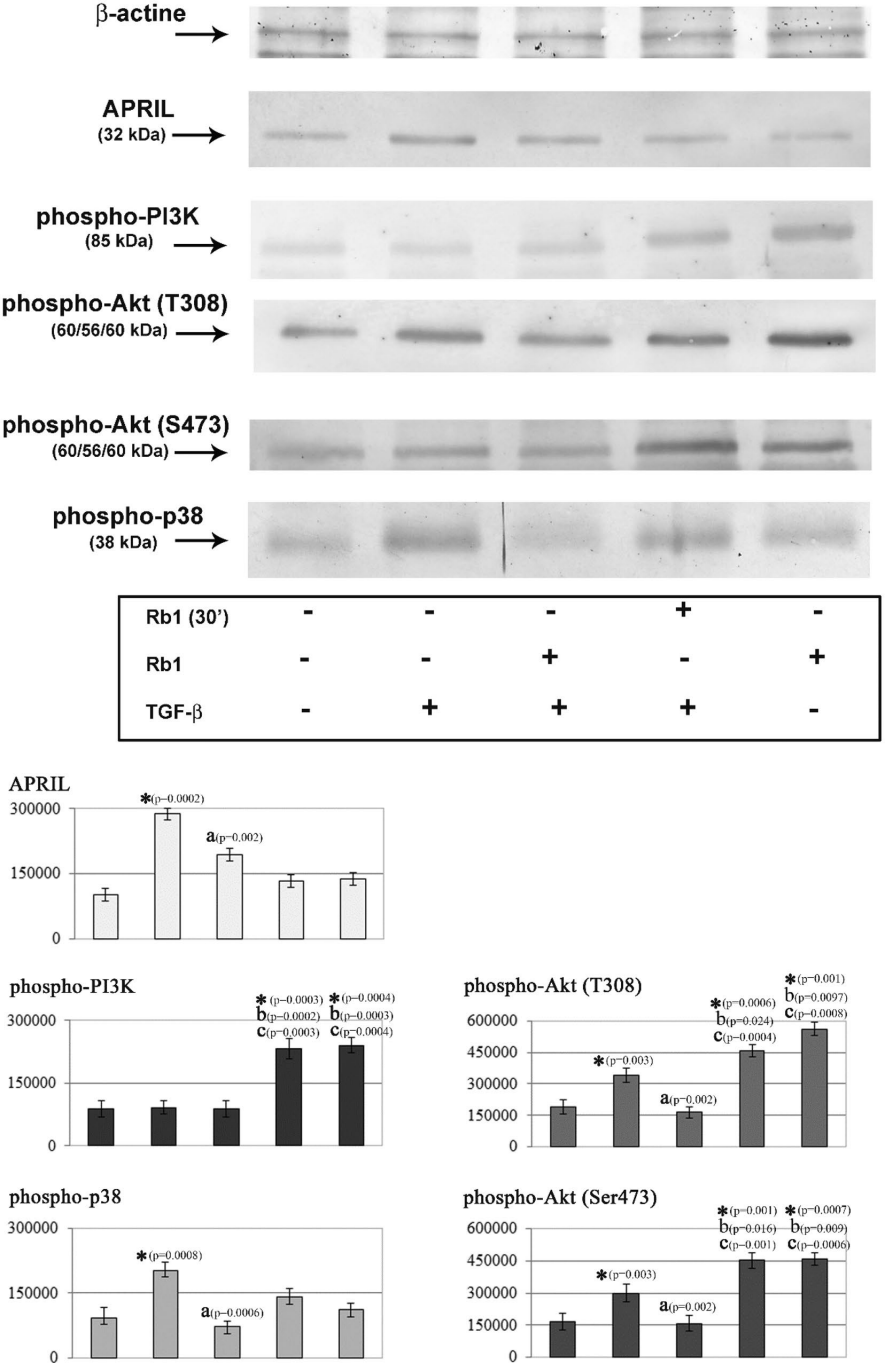
Representative western blots showing the expressions of APRIL, phospho-p38, phospho-PI3K, phospho-Akt(T308) and phospho-Akt(S473) in neutrophils. Neutrophils were treated with TGF-β (10 ng/ml) or with/without Rb1 (50 µg/ml). Cytoplasmic fractions obtained from those cells were used for assessment of APRIL, phospho-p38, phospho-PI3K, phospho-Akt(T308) and phospho-Akt(S473) protein levels via Western blot analyses. Band intensity was quantified using ImageJ software and expressed in arbitrary units (A.U.). Data shown are mean (± SE) of five independent experiments. Value significantly different between * unstimulated and stimulated cells (p < 0.05); **a** cells incubated only with TGF-β and cells incubated with TGF-β and Rb1 (p < 0.05); **b** stimulated cells and incubated only with TGF-β (p < 0.05); **c** stimulated cells and incubated with TGF-β and Rb1 (p < 0.05)

Analysis of the results demonstrated that the exposure of neutrophils to TGF-β increased the expression of APRIL in the cytoplasmic fraction as compared to nonstimulated cells. In addition, these cells showed a higher level of phosphorylation for the following kinases: p38, Akt(T308), and Akt(S473). On the other hand, the phosphorylation of PI3K remained at the same level as in the cells not exposed to TGF-β (Fig. 3).

Incubation of neutrophils with TGF-β and Rb1 reduced the expression of APRIL, as well as the level of phospho-p38, phospho-Akt(T308), and phospho-Akt(S473) in the cytoplasmic fraction, compared to cells stimulated solely with TGF-β. However, no changes were found in the phospho-PI3K level in the cytoplasmic fraction of these cells (Fig. 3).

In addition, no changes were observed in the expression of APRIL and the level of p-p38 in the neutrophils incubated with Rb1 alone, as well as in those incubated first with Rb1 and then with TGF-β, in comparison with the nonstimulated cells. However, a higher level of phosphorylation of the following kinases was seen in these cells: Akt(T308), Akt(S473), and PI3K. The level of phosphorylation was also higher in these cells in comparison with the cells stimulated solely with TGF-β and those incubated first with TGF-β and then with Rb1 (Fig. 3).

**Fig. 4:**
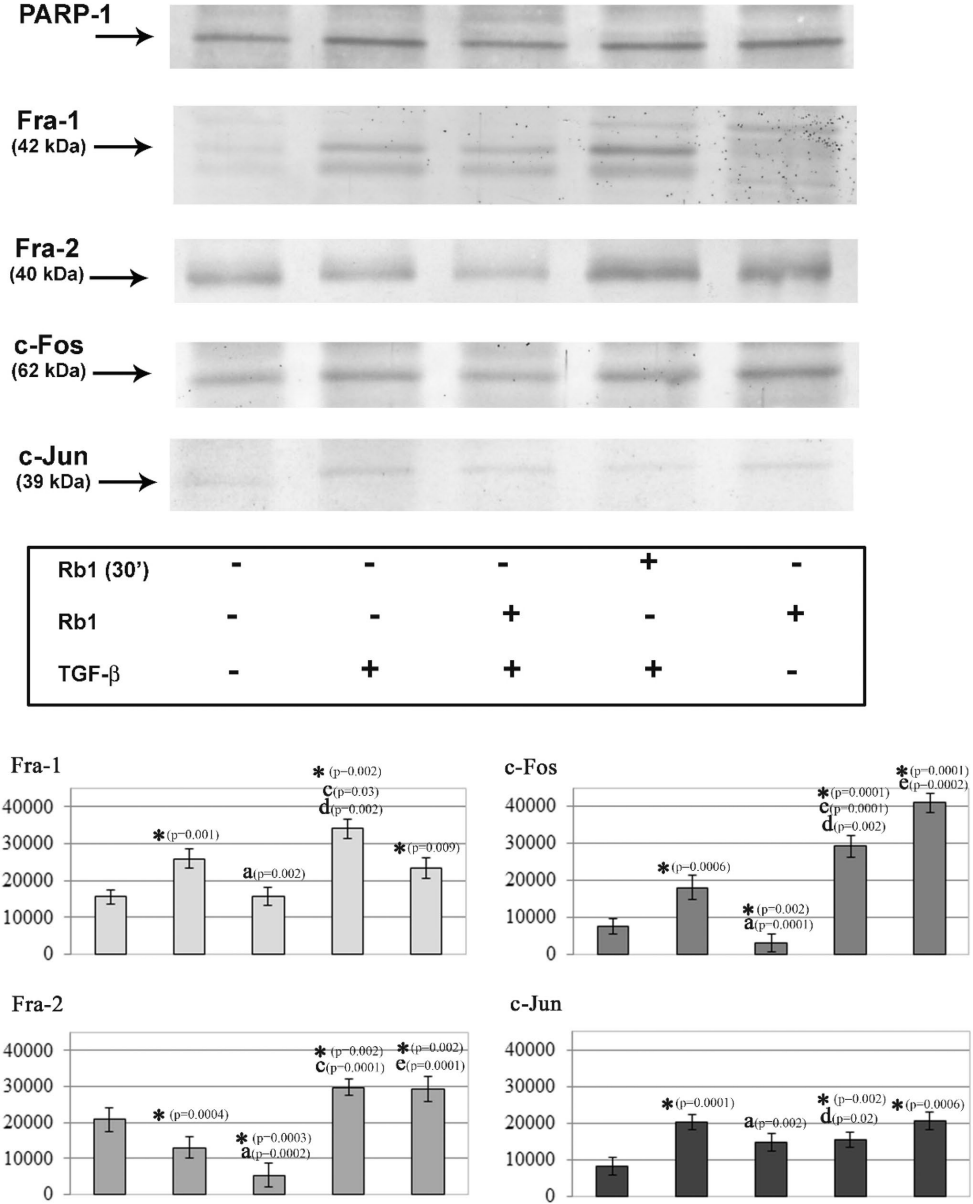
Representative western blots showing the expressions of Fra-1, Fra-2, c-Jun and c-Fos in neutrophils. Neutrophils were treated with TGF-β (10 ng/ml) or with/without Rb1 (50 µg/ml). The nuclear fractions obtained from those cells were used to detect for Fra-1, Fra-2, c-Jun and c-Fos protein levels by Western blot analyses. Band intensity was quantified using ImageJ software and expressed in arbitrary units (A.U.). Data shown are mean (± SE) of five independent experiments. Value significantly different between * unstimulated and stimulated cells (p < 0.05); **a** cells incubated only with TGF-β and cells incubated with TGF-β and Rb1 (p < 0.05); **c** stimulated cells and incubated with TGF-β and Rb1 (p < 0.05); **d** cells incubated only with Rb1 and cells incubated with Rb1 (30’) and TGF-β (p < 0.05); **e** cells incubated only with Rb1 and cells incubated only with TGF-β (p < 0.05)

The neutrophils exposed to TGF-β showed a simultaneous increase in the expression of Fra1, c-Fos, and c-Jun in the nuclear fraction compared to the nonstimulated cells. However, a lower expression of Fra2 was observed in these cells (Fig. 4).

The expression of all the studied transcription factors in the nuclear fraction was lower in the neutrophils incubated with TGF-β and Rb1 compared to the cells incubated only with TGF-β. Additionally, the expression of Fra2 and c-Fos in the studied fraction of these cells was lower compared to that observed in the nonstimulated cells (Fig. 4).

The cells stimulated with Rb1 showed an increased expression of all the studied transcription factors compared to the nonstimulated cells. The expression of Fra2 and c-Fos was also higher in these cells compared to the cells incubated with TGF-β (Fig. 4).

The neutrophils incubated first with Rb1 and then with TGF-β showed a higher expression of Fra1 and a lower expression of c-Fos and c-Jun compared to the nonstimulated cells and the cells stimulated only with Rb1. However, no changes in the expression of the transcription factor Fra2 were found in these cells (Fig. 4).

The expression of Fra1, Fra2, and c-Fos proteins in the cells incubated first with Rb1 and then with TGF-β was higher compared to the cells stimulated first with TGF-β and then with Rb1. However, no changes in the expression of c-Jun were found in these cells (Fig. 4).

## Discussion

To a large extent, the development of cancer is determined by the interaction of cancer cells and cells of the immune system. The effect of their activity is, for example, synthesis and release of cytokines that affect their mutual interactions. The results of many clinical and experimental studies have shown that neutrophils, the cells which constitute the highest percentage among all leukocytes infiltrating the tumor, show a high expression of APRIL in cancer patients (Coffelt et al. 2016; Moreaux et al. 2009).

The results of this study demonstrated for the first time that TGF-β induces the activation of APRIL in human neutrophils through its effects. Changes observed in the level of Akt kinase phosphorylation with the lack of changes in the phosphorylation of the PI3 kinase regulatory subunit p85 suggest that the TGF-β signaling pathway most likely activates Akt kinase by activating another subunit of the PI3 kinase dimer.

Few reports suggest that in human neutrophils, Akt kinase occurs as a complex with p38, MAPKAPK-2, and Hsp27 proteins, which participates in the phosphorylation of T308 and S473, and thus in the activation of AKT, as well as their autophosphorylation leading to the release of these proteins from the complex (Hanada et al. 2004; Hawkins et al. 2010; Okkenhaug 2013). Therefore, elevated phosphorylation of p38 observed in the neutrophils stimulated with cytokine suggests the involvement of p38 kinase in the TGF-β signaling pathway in these cells.

Research data indicate that the activity of proteins forming the transcription factor AP-1 can be regulated at transcriptional and posttranscriptional levels as well as by the phosphorylation of its components by different MAP kinases. It should be emphasized that although p38 does not activate the AP-1 proteins directly, this kinase may regulate the transcription of jun and fos by the phosphorylation of ATF-2, ELK-1, SAP-1, and CCAAT factors, with which it binds to the promoter fragment of jun and fos, which leads to the regulation of their transcription (Karin 1995; Reddy and Mossman 2002; Shaulian and Karin 2002). The obtained data may explain the simultaneous increase in phosphorylation of p38 and expression of transcription factors (c-Jun, c-Fos and Fra-1) in TGF-β-stimulated neutrophils.

Our study also tried to answer question whether demonstrated effect of TGF-β on human neutrophils inducing APRIL expression in these cells, as well as the demonstrated TGF-β signaling pathway, can be regulated by the action of ginsenoside Rb1.

The results of this study showed for the first time that Rb1 decreases the expression of APRIL as well as that of all the signaling proteins in TGF-β-stimulated cells, which indicates that this ginsenoside may modulate the signal activated by these cytokines before the integration of the signal from receptors and tyrosine cytoplasmic kinases, which, with the participation of Ras protein, is then transmitted to the MAP kinase cascade.

Furthermore, our laboratory study with Rb1 showed that this compound does not induce the expression of APRIL and level p38 kinase, whereas it contributes to an increased activation of the PI3K/Akt pathway and all the transcription factors studied.

A similar direction of changes was observed in the cells first treated with Rb1 and then stimulated with TGF-β. Based on the changes observed in the expression of the proteins studied, it can be finally assumed that Rb1 prevents the activation of the signal pathway induced by TGF-β.

The changes in the activity of the PI3K/Akt pathway in neutrophils observed in our study as a result of Rb1 activity may result from the structure of ginsenoside. This compound is classified among phytoestrogens, which are polyphenol nonsteroidal plant compounds with similar biological activity as estrogens (Kang and Min 2012; Xin et al. 2019). The biological action of estrogen is carried out by two different intracellular receptors, the estrogen receptor (ER) α and the ERβ, which are encoded by different genes (DeMayo et al. 2002; Yaşar et al. 2017). It has been shown that there is a direct interaction between ERα and the PI3K pathway that defines the physiological nonnuclear signaling pathway of action of estrogen (Simoncini 2000). According to the reports of other authors, Rb1 augments the cellular antioxidant defenses through ER-dependent HO-1 induction via the Gβ1/PI3K/Akt-Nrf2 signaling pathway, thereby protecting cells from oxidative stress (Hwang and Jeong 2010).

Similar observations were made by other researchers, including Lan et al. (2011), Jeong et al. (2014), and Wang et al. (2008), who concluded that the compound Rb1 exerts a protective effect on the cells by activating the PI3K/AKT pathway.

The results observed regarding the level of Akt phosphorylation with the use of TGF-β and Rb1 in different combinations suggest that these stimulators have a mutually additive effect.

In our research model, the different activities of the effector proteins, which were observed depending on the activation status of neutrophils, indicated a dual mechanism of action of Rb1. In addition, there are data indicating the pro- and anti-inflammatory characteristics of Rb1. In their research on macrophages, Liou et al. (2006) showed that Rb1 may contribute to intensifying the production of inflammatory cytokines. In contrast, Park et al. (2012) demonstrated that Rb1 led to a decrease in cytokine production in BV2 microglial cells and primary cultured microglia stimulated with LPS.

In summary, the study shows that in human neutrophils TGF-β activates the Akt and p38 kinases, and the transcription factors c-Jun, c-Fos and Fra-1, which may be involved in the regulation of APRIL synthesis. However, the Rb1 ginsenoside reduces the effect of TGF-β on APRIL production by resolving the cytokine signaling pathway. Ginsenoside Rb1 appears to be a favorable factor that may be potentially used in the modulation of tumor-promoting APRIL expression.

## Data Availability

The datasets used and/or analyzed during the current study are available from the corresponding author on reasonable request.
